# Dietary Antioxidants Influence IER5 Activation and DNA Repair: Implications for Radioprotection and Healthy Aging

**DOI:** 10.3390/antiox14111357

**Published:** 2025-11-13

**Authors:** Petr Novotný, Ivana Laknerová, Milan Jakubek, Jana Petrusová

**Affiliations:** 1 BIOCEV, First Faculty of Medicine, Charles University, Průmyslová 595, 252 50 Vestec, Czech Republic; novotny@essenceline.cz (P.N.); milan.jakubek@lf1.cuni.cz (M.J.); 2 Essence Line s.r.o., Radiová 1285/7, 102 00 Prague, Czech Republic; 3 Czech Agrifood Research Center, Radiová 1285/7, 102 00 Prague, Czech Republic; ivana.laknerova@carc.cz; 4 Institute of Molecular Genetics, v.v.i., Czech Academy of Sciences, Vídeňská 1083, 142 20 Prague, Czech Republic

**Keywords:** radioprotective compounds, antioxidants, quercetin, curcumin, lycopene, resveratrol, IER5, DNA repair, healthy aging, Czech population

## Abstract

Radioprotective agents derived from natural food sources represent promising candidates for reducing the harmful effects of ionizing radiation and supporting healthy aging. In this study, we investigated the effects of selected micronized bioactive compounds and their mixes on DNA damage response pathways in human retinal epithelial cells (hTERT-RPE1). Individual compounds and their combinations were applied to cultured cells, and the expression of IER5, a radiation-inducible gene associated with DNA repair and cell survival, was evaluated, showing that most potent compound to be lycopene and quercetin. Thus, in the next step, commonly consumed foods available on the Czech market rich in moth—tomato and garlic—were analyzed for their antioxidant capacity. The results revealed marked variability in antioxidant potential among food sources, with specific cultivars exhibiting significantly higher values. Importantly, experimental mixtures of pure and micronized compounds demonstrated distinct and sometimes opposing effects on IER5 expression. These findings indicate that the radioprotective activity of dietary antioxidants depends not only on the properties of individual compounds but also on their specific combinations. Our study provides evidence that phytochemicals such as quercetin, lycopene, but also partially resveratrol and curcumin can modulate DNA-repair-associated pathways and underscores their potential as combinatory agents in strategies aimed at promoting genomic stability and potentially healthy aging.

## 1. Introduction

Ionizing radiation, especially highly penetrating gamma rays, represents a significant biological stressor with a broad spectrum of harmful effects on living cells. By virtue of its high energy, gamma radiation can penetrate deeply into tissues and interact with water molecules, lipids, proteins, and DNA [1,2,3]. These interactions cause direct damage to genetic material and also produce reactive oxygen species (ROS) through radiolysis of water. The ROS (such as hydroxyl radicals and peroxides) inflict secondary oxidative injuries to cellular components. A particularly important type of damage from ionizing radiation is DNA double-strand breaks (DSBs), which are widely regarded as one of the most severe forms of cellular damage [1,3]. Unrepaired or misrepaired DSBs can destabilize the genome and lead to mutations, cell cycle disturbances, or trigger apoptosis [4,5]. If the radiation-induced damage exceeds the cell’s repair capacity, the result is often cell death (via apoptosis or necrosis) or potentially carcinogenic transformations in surviving cells [5,6,7].

Certain tissues are especially sensitive to ionizing radiation, particularly those with high mitotic activity and oxygenation. Rapidly proliferating tissues like the bone marrow, gastrointestinal epithelium (including oral mucosa), and skin are among the most radiosensitive organs [8,9]. In these tissues, radiation exposure impairs the normal regenerative capacity, leading to acute toxicity symptoms. Clinically, this is evident in, for example, skin inflammation and erythema progressing to dry or moist desquamation, and even the ulceration of irradiated areas [8]. Likewise, damage to intestinal crypt cells can cause mucosal ulcerations, nausea, and diarrhea, and disruption of hematopoietic stem cells in bone marrow results in cytopenia and systemic inflammatory responses at higher doses. Such acute radiation injuries reflect the inability of these high-turnover tissues to repair damage quickly enough, underscoring the need for protective strategies to safeguard normal cells during radiation exposure.

One approach to limiting the harmful effects of radiation on healthy cells is the use of radioprotective agents. These are natural or synthetic compounds capable of protecting cells from ionizing radiation damage through various mechanisms [10,11,12]. Notably, many radioprotectors act as antioxidants that scavenge free radicals, thereby reducing the ROS-mediated component of radiation injury [11]. Some compounds can suppress the initial formation of free radicals or accelerate their removal, whereas others boost the cell’s own antioxidant defenses by up-regulating enzymes like superoxide dismutase, catalase, and glutathione peroxidase [11]. Additional radioprotective mechanisms include stabilizing DNA and cellular membranes to prevent damage, enhancing DNA repair pathways, and modulating cellular stress responses [12]. For example, certain radioprotectors may activate cell survival signaling or delay cell-cycle progression, giving cells more time to repair DSBs before dividing [1,13]. There is also evidence that some agents can influence apoptosis pathways or induce cytoprotective proteins; in other words, they might decrease unwarranted apoptotic death in normal cells while promoting repair, or conversely, ensure damaged cells undergo apoptosis to avoid malignant transformation. Overall, multiple mechanisms often act in concert, and an ideal radioprotective compound would combine antioxidant, anti-mutagenic, and pro-recovery activities [14]. Several such compounds have shown promise in preclinical studies and are being investigated for their ability to reduce radiation toxicity in medical and environmental exposure scenarios [15,16].

In this project, we focus on evaluating the radioprotective potential of selected natural compounds: quercetin, curcumin, betacarotene, lutein, lycopene, and kelp (extracts from brown seaweeds). Many of these substances are well known for their strong antioxidant and anti-inflammatory properties. Importantly, these compounds originate from common dietary sources, tend to have low toxicity and good bioavailability at feasible doses, and thus lend themselves to potential use as dietary supplements or food-based interventions for radioprotection. An attractive aspect of natural radioprotectors is the possibility of combining them into complex mixtures (e.g., antioxidant-rich diets or nutraceutical formulations) that could act synergistically to enhance protection [17,18]. This multi-faceted strategy—using a mixture of radioprotective nutrients—might better mimic real-world dietary patterns and cover a broader range of protective mechanisms, from ROS scavenging to inflammation modulation.

The considerations above formed the basis of our experimental design. We set out to prepare and test both the individual compounds and various blended mixtures of these radioprotective agents formulated to resemble combinations found in normal foods or diets. Our aim was to systematically and quantitatively evaluate the capacity of these substances (alone and in mixture) to protect cultured cells from radiation injury. In our model, a stable human cell line was exposed to a defined sublethal dose of gamma radiation, with or without pretreatment by the radioprotective compounds or their mixtures. We focused on several key endpoints to assess radioprotection efficacy: the extent of DNA damage after irradiation (particularly DNA double-strand breaks, measured by appropriate markers), the ability of cells to recover and proliferate following radiation exposure, and overall cell viability/survival when returned to standard culture conditions. These measures were chosen to capture both the immediate molecular damage (DNA integrity) and the longer-term functional outcome (cell survival and regrowth). By comparing treated versus untreated cells, we can determine the radioprotective efficacy of each agent and of the combined mixtures.

Our results will deepen our understanding of cellular responses to radioprotective treatments and the mechanisms by which these natural compounds mitigate radiation injury. Ultimately, this research can contribute to the development of practically applicable preventive measures to guard against radiation damage in both clinical and civil contexts. For instance, positive findings could inform nutritional recommendations or supplement formulations for individuals undergoing radiotherapy (to protect normal tissues) or for populations at risk of radiation exposure (such as nuclear industry workers or in emergency scenarios). In summary, by exploring both single compounds and synergistic mixtures, our study aims to identify effective radioprotective strategies that are grounded in readily available natural substances and supported by scientific evidence on their protective benefits. The knowledge gained could pave the way toward safer radiation use in medicine and improved preparedness against radiological hazards in the environment.

## 2. Materials and Methods

### 2.1. Radioprotective Compounds

Selected supplements used in this project were quercetin, betacarotene, curcumin, resveratrol, lycopene, lutein, and a kelp extract. Each compound was chosen on the basis of its pronounced antioxidant capacity and documented ability to moderate oxidative or inflammatory stress, properties that are directly relevant to the protection of nuclear integrity under ionizing radiation. All substances were procured as commercially available, food-grade dietary supplements manufactured to pharmaceutical-quality standards (Table S1). Most of the product was supplied as a single-component formulation with a declared content of the active substance enabling accurate dosage for cell-culture experiments. Only the kelp extract was used as a mixture with the approximate dosage recommendation according to the dosage of iodine.

### 2.2. Micronization by Nanomilling

Prior to experimental use, each compound was micronized with a planetary nano-ball mill to obtain sub-micron particle sizes, thereby improving both biological availability and suspension homogeneity [19,20]. Capsules or tablets were first mechanically comminuted and then milled in an E-max high-energy ball mill (Retsch, Haan, Germany) equipped with 20 g of grinding beads. Milling was carried out for 15 min at 1000 Hz. Particle-size measurements were performed on a SALD laser diffraction analyzer (Shimadzu, Kyoto, Japan).

### 2.3. Stock Solutions of Radioprotective Compounds

The preparation of stock solutions of individually tested radioprotective agents, which were used for application to cell cultures, included a concentration that has been verified as non-toxic to cultured cells but with antioxidant potential. Each dilution was selected based on available scientific studies demonstrating its strong antioxidant or protective effects against oxidative stress and DNA damage (Table S2).

Stock solutions were prepared from micronized supplements dissolved in phosphate-buffered saline (PBS), a commonly used buffer in biological and biochemical applications. PBS contains sodium chloride (NaCl), potassium chloride (KCl), sodium dihydrogen phosphate (NaH_2_PO_4_), and disodium hydrogen phosphate (Na_2_HPO_4_), which together maintain a stable pH of approximately 6.0–7.4, essential for cell culture experiments. For lipophilic supplements such as betacarotene, lycopene, and lutein, Tween 20 was added to the solution at a final concentration of 0.1% to prevent phase separation and precipitation of the supplement without causing any damage to the cells.

The recommended concentration applied to cells takes into account the physiological efficacy of the substance, solubility, stability under culture conditions, and potential toxicity. The final volumes applied to individual wells (1 mL of medium + corresponding volume of substance) were optimized to ensure consistent dosing between tests. This standardized approach enabled reproducible application of all substances across experiments and also created a basis for evaluating their potential to protect cells from the effects of ionizing radiation.

### 2.4. Cell Line and Induction of DNA Damage by Irradiation

For our experiments, we used hTERT RPE-1 cell line (Thermo Fisher Scientific, Waltham, MA, USA), which is an immortalized somatic line of retinal pigment epithelial cells. This line is commonly used for testing and quality control in drug development, is well characterized, and is easy to cultivate. We maintained the cells in Dulbecco’s Modified Eagle Medium (DMEM, high-glucose formulation, 4.5 g/L; Gibco, Frederick, MD, USA), a widely used basal medium designed to support the growth and proliferation of diverse mammalian cell types. The formulation provides essential and non-essential amino acids—including glycine, L-glutamine, and tyrosine—together with vitamins and cofactors such as folic acid, biotin, pantothenate, niacin, inositol, and choline that are required for membrane biosynthesis and general cellular metabolism. DMEM was supplemented with 10% (*v*/*v*) heat-inactivated fetal bovine serum (FBS) and a standard antibiotic mixture of penicillin (100 U/mL) and streptomycin (100 µg/mL). Cultures were incubated at 37 °C in a humidified atmosphere of 5% CO_2_ and sub-cultured when they reached approximately 70–80% confluency. The cells were co-cultivated with radioprotective agents 24 h before irradiation. All further experiments proceeded 3 h post-irradiation. DNA damage induced by gamma radiation was performed on cell lines cultured under standard good laboratory practice conditions. This cell line is routinely used in irradiation studies, where the dose varies but is mostly applied reaching up to 20 Gy, applied as a sublethal dose [21,22].

### 2.5. Microscopy

Live-cell imaging of co-cultured cells was performed using a Leica DM6000 inverted microscope (Leica Microsystems, Wetzlar, Germany), equipped with an incubation chamber maintained at 37 °C and 5% CO_2_. Imaging was conducted 48 h after co-cultivation of the cells with the tested compounds, using a bright-field HCX PL APO 40×/0.75 air objective. Cells treated only with the buffer used for compound solubilization (PBS with 0.1% Tween 20) served as the control. The acquired images were processed and analyzed using ImageJ software (version 1.41) [23].

### 2.6. RNA Isolation and qPCR

**Figure 1:**
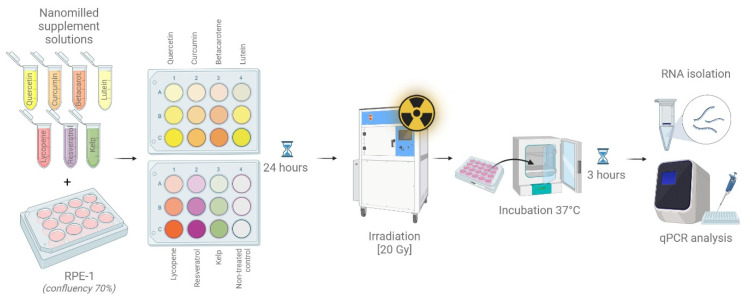
Graphical manual showing experimental pipeline testing micronized supplements on RPE-1 cell line to test their radioprotective potential.

Total RNA was extracted from cells using an RNAeasy RNA Extraction Kit (Qiagen, Hilden, Germany). Reverse transcription (RT-PCR) was performed using a Thermo Scientific™ RevertAid RT Reverse Transcription Kit (Fisher Scientific) according to the manufacturer’s instructions. Quantitative PCR (qPCR) was then performed using SYBR Green Real Master Mix (TIANGEN Biotech Beijing Co., Ltd., Beijing, China). The following primers were used for expression detection: IER5 (Gene ID: 51278; Fw: 5′CCGGGAACGTGGCTAACC3′; Rev: 5′TTCCGTAGGAGTCCCGAGAA3′), γH2AX (Gene ID: 3014; Fw: 5′CGGCAGTGCTGGAGTACCTCA 3′; Rev: 5′AGCTCCTCGTCGTTGCGGATG3′), and the reference gene β-actin (Gene ID: 60; Fw: 5′GCGCGCTACAGCTTCA3′; Rev: 5′CTTAATGTCACGACGATTTCC3′). Relative quantification of IER5 as well as γH2AX gene expression was evaluated in relation to the stably expressed control gene β-actin. RNA isolation and expression analysis were performed three hours after irradiation of cell cultures. For the overview of the methodological pipeline, please see illustration at the Figure 1.

### 2.7. Testing of Antioxidant Activity

Tested antioxidant activity of selected plant materials—containing lycopene and quercetin—was assessed using two complementary and widely applied methods: DPPH and FRAP. These assays are based on distinct analytical principles. The DPPH method measures radical scavenging capacity, utilizing the stable radical 2,2-diphenyl-1-picrylhydrazyl. Upon reaction with antioxidants, DPPH undergoes a measurable color change, with a decrease in absorbance at 515 nm recorded at a fixed time. In contrast, the FRAP assay quantifies the ferric reducing antioxidant potential of a sample: antioxidants reduce the Fe^3+^–TPTZ (2,4,6-Tris(2-pyridyl)-s-triazine) complex to Fe^2+^–TPTZ, producing a color change measurable as an increase in absorbance at 593 nm.

## 3. Results

### 3.1. DNA Damage Induced by Gamma Radiation and Radioprotective Defense

Gamma radiation, a highly energetic form of ionizing radiation, can cause severe DNA damage both directly—through double-strand breaks (DSBs)—and indirectly via reactive oxygen species (ROS) generated by water radiolysis [1]. These radicals attack DNA, proteins, and membrane lipids, amplifying cellular injury and oxidative stress [15,24]. Although cells activate repair pathways, excessive damage may overwhelm these systems, resulting in apoptosis or loss of genomic stability [25]. Although repair systems are activated, excessive damage may exceed repair capacity. Tissues with rapid cell turnover—such as skin, intestinal mucosa, and bone marrow—are particularly sensitive [26,27]. These effects underscore the need for radioprotective agents capable of reducing oxidative and genotoxic injury in normal cells.

This study focused on formulating and testing mixtures of natural antioxidants available on the market to determine their ability to mitigate γ-induced cellular damage in vitro. Notably, the cells used in this study are known to be less sensitive to irradiation and require either higher dosage or smaller but chronic doses (e.g., 20 mGy/1 h) of irradiation under experimental conditions. Lower doses were previously reported to have no effect on the DNA integrity of RPE-1 cells; therefore, we did not test different doses and applied the sublethal dosage, as confirmed elsewhere [28].

### 3.2. Protective Potential of Selected Natural Compounds Against Gamma Radiation

Radioprotective agents—whether synthetic or natural—act mainly by scavenging ROS, enhancing endogenous antioxidant defenses, stabilizing DNA, and promoting repair [29]. We picked seven natural compounds with documented antioxidant or anti-inflammatory activity.

Quercetin is a flavonol abundant in onions, apples, and berries. In erythrocytes exposed to 2 Gy γ-rays, quercetin pretreatment preserved glutathione, reduced lipid peroxidation, and prevented hemolysis, demonstrating a clear ROS-scavenging and membrane-stabilizing effect [30,31,32,33,34].

A provitamin-A carotenoid abundant in carrots and sweet potatoes quenches singlet oxygen and reduces γ-ray-induced chromosomal aberrations [33,34]. Its action is linked to membrane radical scavenging and support of DNA-repair enzymes [35,36].

Curcumin (the yellow pigment of *Curcuma longa*) simultaneously suppressed NF-κB activation, up-regulated superoxide–dismutase and catalase, and decreased γ-H2AX formation across multiple tumor and normal cell lines. Animal studies show improved survival of irradiated rodents and reduced radiotherapy-induced dermatitis [20,37]

Resveratrol is a stilbene polyphenol that enhances glutathione and SOD activity while quenching hydroxyl radicals. Pre-irradiation treatment in animals reduces bone-marrow damage and improves survival via p53 and Bcl-2 modulation [38,39].

Lycopene, the red carotenoid of tomato, watermelon, and grapefruit, is a red carotenoid found in tomatoes and watermelon; one of the most efficient singlet-oxygen quenchers. Pretreatment reduces γ-ray-induced DNA strand breaks and normalizes antioxidant balance [40]. High dietary intake correlates with lower cancer risk.

Lutein, a xanthophyll concentrated in leafy greens and in the retinal macula, absorbed high-energy blue light and reduced UV-induced ROS. However, the facts about irradiation protection of Lutein remain limited only to mouse tissues [40].

The last compound—Kelp—usually refers to brown seaweeds like kombu (*Saccharina*/*Laminaria*), sugar kelp (*Saccharina latissima*), and related species. They’re notable for very high iodine and useful polysaccharides (e.g., fucoidan). Iodide accumulation in kelp tissues functions as an inorganic antioxidant, a role it also fulfils in mammalian plasma [41].

Collectively, these natural compounds represent a diverse spectrum of antioxidant and anti-inflammatory agents capable of limiting γ-radiation-induced oxidative and genotoxic stress through complementary mechanisms. Their protective efficiency, however, depends not only on intrinsic chemical reactivity but also on biological accessibility within target tissues. Since most of these phytochemicals are poorly soluble and exhibit limited absorption in their native forms, improving formulation and delivery becomes essential to fully exploit their radioprotective potential. This aspect is further addressed in the following section on micronization and its impact on bioavailability.

### 3.3. Micronization of the Compounds Increases Biological Availability

Because each selected phytochemical is hydrophobic to some extent, its effectiveness in vitro and in vivo is often compromised by poor aqueous solubility and slow dissolution. Reducing the primary particle size to the sub-micron or nano domain substantially increases specific surface area, shortens diffusion paths, and enhances wetting—thus accelerating dissolution and, consequently, boosting cellular uptake and systemic exposure. This micronization step is critical for improving solubility, dissolution rate, and absorption of dietary supplements, thereby amplifying their therapeutic potential.

Specifically, each compound in our study has previously been shown to benefit from particle-size reduction. Quercetin, for example, when formulated into nano-emulsions, demonstrated increases in dissolution, permeability, and oral bioavailability by approximately 11.7-, 3.4-, and 28.3-fold, respectively, compared with unformulated quercetin [42]. Betacarotene, while having fewer direct micronization-specific studies, is known to benefit from particle size reduction, with improved solubility and consistency enhancing its bioavailability and efficacy as a fat-soluble antioxidant. Curcumin has been extensively studied in nanotechnology-based delivery systems, including polymeric, lipidic, and micellar formulations, which improve its solubility, stability, and bioavailability—substantially enhancing its pharmacokinetics [43,44]. Lycopene, though having fewer dedicated micronized studies, has shown improved dissolution and biological accessibility in nano-formulated carotenoids, supporting its antioxidant role. Micronized resveratrol (SRT501) has demonstrated a 3.6-fold increase in plasma concentration compared with non-micronized forms, confirming enhanced pharmacokinetics [45]. Lutein is commonly formulated in micronized forms within supplements to ensure consistent dosing and improve absorption, particularly in the management of age-related macular degeneration. Kelp extract, although lacking direct micronization trials, benefits from fine processing to deliver uniform iodine doses and improve bioavailability, mitigating the variability of raw kelp preparations in nutritional applications.

**Figure 2:**
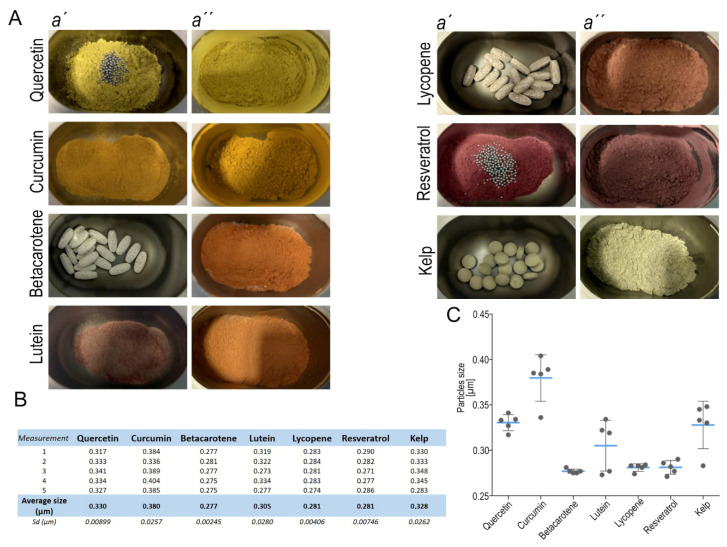
(**A**) Preparation of supplements before (**a′**) and after nanomilling (**a″**); (**B**) table of particle sizes of individual nanomilled supplements in five independent measurements and the average value of the contained particles; (**C**) particle size distribution of the micronized compounds. The graph illustrates the consistency of particle sizes for each supplement. Statistical evaluation of replicate measurements revealed no significant deviations (all *p* > 0.05), confirming uniform and reproducible particle size within each formulation.

Guided by these observations, we subjected all micronized seven radioprotective agents to high-energy planetary nanomilling prior to cell-culture experiments. Either whole tablets or decapsulated compounds were transferred into the milling bowl (Figure 2A). The milling parameters were optimized to yield narrow, sub-micron-sized distributions while avoiding thermal degradation or polymorphic transitions. After dispersion by brief probe sonication, the nano-suspensions remained visibly stable for at least 4 h at 37 °C, indicating adequate colloidal homogeneity for subsequent irradiation assays. Post-milling average diameters ranged from 0.277 µm to 0.380 µm (Figure S1). Betacarotene yielded the smallest average particles (0.277 µm), whereas curcumin exhibited the largest (0.380 µm) (Figure 2B,C). These dimensions were considered suitable for in vitro testing because they facilitate efficient cellular uptake while minimizing the risk of agglomeration. Individual size distributions are provided in the accompanying measurement summary. Measurement record after milling shows a range of particles sizes (Figure S1). Measurement was repeated five times for better accuracy.

### 3.4. Physiological Effect of Compounds on the Cells

**Figure 3:**
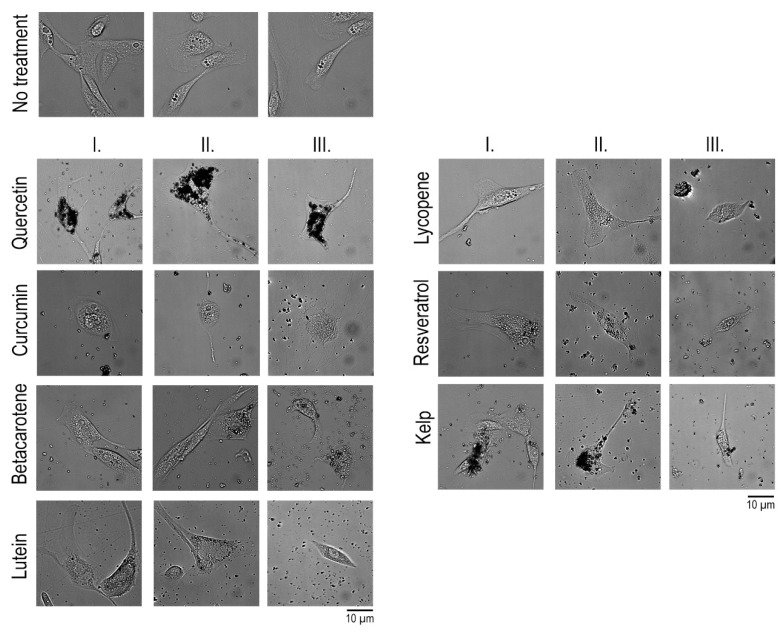
Imaging of live cells co-cultivated with radioprotective supplement. Upper panel shows cells without treatment (three positions on the petri dish); column I. contained recommended dose of the supplement, column II. contained a tenfold higher amount of supplement, column III. contained a twentyfold higher amount of supplement.

We co-cultured hTERT RPE-1 cells with selected radioprotective supplements in three different concentrations corresponding to: (I) the recommended application dose according to the literature, (II) ten times higher concentration, and (III) twenty times higher concentration (Table S2). The aim was to monitor any morphological changes and cytotoxic effects depending on the concentration of the substance. After incubation, we performed a visual assessment of their morphology. We focused on the presence of signs of stress, such as shape change, loss of adhesion, or vacuolization, which would indicate a negative impact of the applied concentrations. We maintained control cells without the addition of supplements in the same culture medium (Figure 3, upper panel) and compared them to supplemented cells. The recommended concentrations of the individual supplements as indicated in Table S2 did not damage the cells in any way, and it was clear that the morphology, condition, and density of the cells corresponded to those of the control cells. In the case of quercetin, it was evident that the cells phagocytosed the substance in the form of concentrated vesicles, which are visible as dense structures (Figure 3, Quercetin, column I., II., and III., and Kelp, column I. and II.), but this did not affect the condition of the cells. Higher-than-recommended doses of some supplements caused cell death and contraction, especially in the case of curcumin (column II. and III.), but also in the case of most other supplements when the concentration was twenty times higher than the recommended dose (column III.).

### 3.5. Assessment of IER5 and γH2AX Activation as an Early Indicator of DNA Damage and Repair

To quantify the extent of γ-ray-induced DNA injury and the ensuing cellular repair response, we measured transcription of the Immediate Early Response 5 (IER5) gene. IER5 belongs to the class of stress-response genes whose expression is rapidly and transiently up-regulated after diverse insults, including ionizing radiation [46]. Induction can be detected within 30–60 min of exposure, and IER5 therefore serves as a sensitive early marker of genotoxic and oxidative stress [47]. Functional studies have linked IER5 to modulation of cell-cycle checkpoints, chromatin remodeling, and non-homologous end-joining repair of DNA double-strand breaks. Its activation is closely associated with phosphorylation of histone H2AX (γH2AX), a canonical marker of double-strand DNA breaks. While γH2AX reflects the direct formation of DNA lesions [48,49], IER5 represents a downstream transcriptional response to this damage, indicating engagement of repair and stress-signaling pathways. The coordinated induction of both markers thus provides complementary information—γH2AX as an indicator of DNA breakage and IER5 as a measure of the cellular reaction to such genotoxic stress.

Our central hypothesis was that an effective radioprotective treatment would prevent or greatly attenuate the induction of primary IER5 but also γH2AX. Suppression of γH2AX formation would indicate that DNA double-strand breaks had been efficiently prevented, while reduced IER5 transcription would suggest that the downstream stress and repair signaling pathways were not activated. Conversely, a pronounced rise in γH2AX together with elevated IER5 expression would imply that irradiation had caused substantial DNA injury, thereby triggering both damage signaling and transcriptional repair responses. Cells were co-cultured with the tested compounds at the recommended viable concentration (I), tenfold higher concentration (II), and twentyfold higher concentration (III). Following co-cultivation, the cells were exposed to a sublethal dose of γ-radiation. Three-hour post-exposure total RNA was extracted and reverse-transcribed; IER5 and γH2AX mRNA were quantified by qPCR and normalized to the housekeeping gene β-actin. Each experiment was performed in duplicate, and every qPCR reaction was conducted in technical triplicate. As shown in Figure 3, each data point represents the mean value of the triplicate measurements from each experimental replicate.

**Figure 4:**
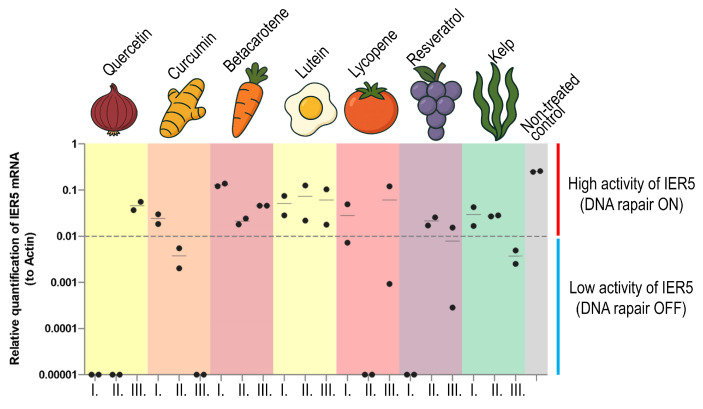
Quantification of IER activity in irradiated cells, supplemented with radioprotective compounds. Each compound depicted by a color box in the graph was added to the cells in recommended concentration (I.), ten times higher (II.) and twenty times higher (III.). Level of IER5 expression was quantified relatively to β-actin as a house-keeping gene. The threshold depicted with the dashed line splits the graph into two sectors—low IER5 activity (bottom, blue line) and high IER5 activity (upper, red line). Median is calculated for each measurement. The gray box of non-treated control shows the highest activity of the IER5 gene observed. For statistical evaluation and data significance, see Table S3.

Marked differences emerged among the compounds. Quercetin, resveratrol, and lycopene suppressed IER5 expression to, or below, the assay’s detection threshold at all three concentrations, implying near-complete protection against γ-induced DNA damage (Figure 4). In contrast, curcumin, betacarotene, and lutein yielded appreciable IER5 induction—particularly at the two lower doses—suggesting that these agents primarily supported repair pathways rather than preventing initial damage. Kelp extract produced a modest but reproducible increase in IER5 (≈0.01 relative units), consistent with partial protection via combined antioxidant and mild repair-stimulating activity. Changes in IER5 expression across different compound concentrations were variable, and statistical analysis revealed that all tested compounds, except lutein, showed a clear concentration-dependent effect. Lutein exhibited only a weak radio-protective effect at all tested concentrations (Table S3). The negative control values remained within the same low radio-protective effect, confirming the validity of the assay (Figure 4).

Comparing of the IER5 expression profile with γH2AX expression profile showed, the expression of IER5 generally followed the pattern of γH2AX accumulation, consistent with its role as a transcriptional target of radiation-induced stress signaling (Figure S2). The detailed relationship between IER5 and γH2AX expression was evaluated by a regression analysis (Table S4 and Figure S3), showing the correlation between IER5 and γH2AX transcript levels (log_10_-transformed relative expression). A moderate positive correlation of all compounds was observed between *IER5* and γH2AX expression (Pearson *r* = 0.42, Spearman *p* = 0.048), indicating that increased transcriptional stress signaling was generally associated with higher levels of DNA double-strand breaks (Figure S3). Compounds such as quercetin and lycopene clustered at the lower expression range, consistent with strong radioprotective effects (Figure S3, lower left quadrant), whereas resveratrol, β-carotene, and kelp showed elevated γH2AX levels, indicating persistent or secondary DNA damage (Figure S3, upper left quadrant). This may be considered as their potential for high radioprotective capacity. In contrast, β-carotene and kelp at higher doses produced disproportionate increases in γH2AX without parallel IER5 induction, suggesting potential pro-oxidant or cytotoxic effects (Figure S3, right part of the plot).

Collectively, the data identify quercetin and lycopene as the most potent radioprotective agents in this model, completely abrogating both IER5 and γH2AX activation, whereas curcumin, lutein, and betacarotene provided only limited or secondary protection. Kelp extract exerted a mild yet consistent mitigating effect on the cellular stress response.

**Figure 5:**
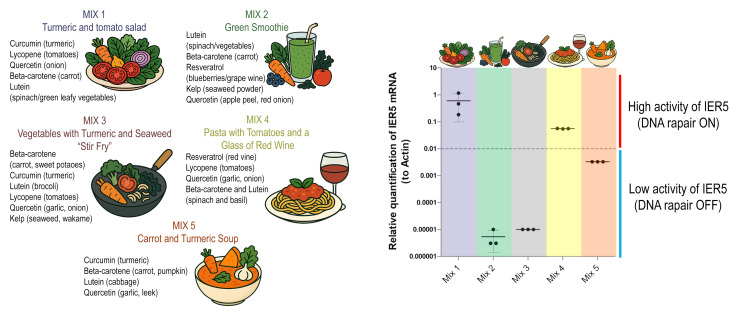
Formulated mixtures of radioprotective compounds and their effects on IER5 expression. (**Left**) Composition of five experimental mixtures (MIX 1–MIX 5) created from defined supplements in proportions resembling common meal-like combinations (e.g., turmeric–tomato salad, green smoothie, stir-fry with seaweed, pasta with tomatoes and wine, carrot–turmeric soup). (**Right**) Relative quantification of IER5 mRNA expression in hTERT-RPE1 cells treated with the mixtures, normalized to β-actin. MIX 1 and MIX 4 markedly induced IER5 expression, consistent with activation of DNA repair pathways. MIX 2, MIX 3, and MIX 5 elicited minimal induction, indicating down-regulation of the DNA repair response. Data points represent 1 independent qPCR assay; bars indicate mean values and standard deviation.

Building on these results, we next aimed to evaluate whether combinations of radioprotective compounds would act synergistically, neutrally, or antagonistically when applied together. To this end, we formulated experimental mixtures containing defined sets of purified supplements, selected based on their individual effects and arranged to resemble common meal-like combinations (Figure 5). Importantly, although their appearance mimicked real dishes, the mixtures were prepared exclusively from supplements dissolved in culture medium and did not contain any whole-food material.

Each mixture (MIX 1–MIX 5) was designed to test different compositional principles: MIX 1 combined turmeric and tomato components, MIX 2 represented a green smoothie-like blend of lutein, carotenoids, and polyphenols, MIX 3 resembled a vegetable–seaweed stir-fry rich in carotenoids and flavonoids, MIX 4 integrated tomato constituents with resveratrol to mimic a pasta and wine pairing, and MIX 5 combined turmeric and carrot components in a soup-like formulation (Figure 5, left panel). When applied to hTERT-RPE1 cells, these mixtures elicited strikingly divergent outcomes in IER5 expression. MIX 1 and MIX 4 induced strong activation of IER5, consistent with robust DNA repair pathway stimulation, whereas MIX 2 and MIX 3 produced only minimal induction. In contrast, MIX 5 markedly suppressed IER5 expression to levels below baseline, effectively switching off the repair response (Figure 5).

These findings demonstrate that the biological outcome of radioprotective agents cannot be predicted solely from their individual properties, as specific combinations may either potentiate or counteract each other’s effects. This underscores the importance of considering interactions within complex mixtures when evaluating the nutritional or therapeutic potential of natural antioxidants.

### 3.6. Evaluation of the Effectiveness of Quercetin and Lycopene in Commonly Consumed Foods Available on the Czech Market

Although our measurements focus on food-derived sources (garlic for quercetin; tomato for lycopene), the antioxidant capacities of the pure compounds are well established to be markedly higher than values observed in complex food matrices. Recent bench studies using standardized DPPH and FRAP assays have consistently placed pure quercetin among the most potent small-molecule antioxidants across tested panels, outperforming other comparators across concentration ranges [50]. Likewise, contemporary work underscores the exceptional radical-quenching capacity of lycopene relative to that of other carotenoids in lipophilic systems, reflecting its strong singlet-oxygen quenching efficiency [51].

Importantly, matrix effects (co-constituents, microenvironment, solubility/bioaccessibility) temper the apparent activity in foods versus the neat standards; interactions within real foods can either diminish or modulate the redox behavior of isolated antioxidants, helping explain why food-based values are usually lower than the pure-compound benchmarks [52]. Although our measurements focus on food-derived sources (garlic for quercetin; tomato for lycopene), the antioxidant capacities of the pure compounds are well established to be markedly higher than those observed in complex food matrices. Recent studies using standardized DPPH and FRAP assays have consistently reported pure quercetin activities in the range of 350–500 mg TE/g, ranking it among the most potent small-molecule antioxidants tested [53,54]. In contrast, antioxidant activity of pure lycopene shows very low activity in standard assays. Pure lycopene’s solubility in polar solvents used for DPPH and FRAP results is underestimating the activity (<0.1 mg TE/g) [55]. In accurate conditions, using lipid-soluble assays (e.g., crocin bleaching or ORAC) for accurate assessment of its lycopene activity shows moderate potency (0.6–1.6 mg TE/g) [56].

In the following section, we focused on the evaluation of antioxidant activity of bio-available forms of radioprotective compounds—quercetin and lycopene—which are available and traditional on the market. The evaluation of tomato and garlic cultivars available on the Czech market highlights not only the variability in antioxidant potential among foods but also their potential relevance for public health. In the next step, we evaluated the antioxidant potential of the food rich in lycopene and quercetin which are available on the Czech market and may be potentially recommended to consumers.

#### 3.6.1. Lycopene in Tomatoes

Lycopene is a carotenoid synthesized by plants and certain microorganisms, found predominantly in red fruits and vegetables, including tomatoes (*Solanum lycopersicum*), watermelon, pink grapefruit, apricots, pink guava, and papaya. Tomatoes are among the most widely consumed sources of lycopene worldwide. In the Czech Republic in 2018, total tomato consumption—including fresh produce for direct sale, processing, and household use—was 11.8 kg per capita per year, approximately twice that of cucumbers or sweet peppers [57]. Tomatoes are consumed both raw and processed, contributing substantially to dietary antioxidant intake. Greenhouse cultivation allows year-round availability of fresh tomatoes. The effect of processing on antioxidant levels in tomatoes has been extensively studied; heat-treated tomatoes contain more bioavailable lycopene than raw tomatoes, as heat disrupts cell structures and promotes lycopene release and absorption. For fresh tomatoes, consumption with skin is recommended, as the majority of lycopene is concentrated in the peel.

**Figure 6:**
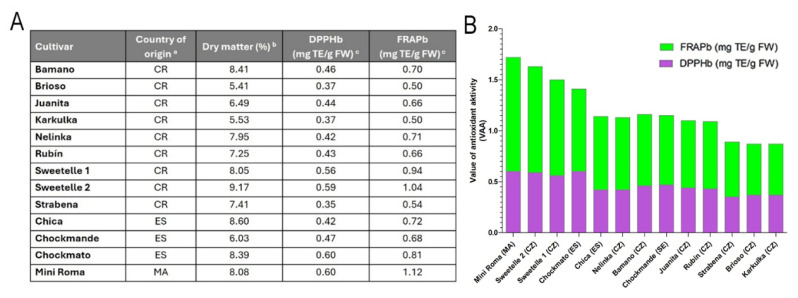
(**A**) Chart showing name of tomato cultivar; (a) country of origin in alpha-code: CR—Czech Republic, ES—Spain, MA—Morocco; (b) dry matter content expressed as % fresh weight; and (c) antioxidant activity expressed as mg Trolox equivalents (TE) per gram of fresh weight (FW). (**B**) Measured values of antioxidant activity cumulated for each cultivar and sorted from the highest to lowest evaluated value. The paired *t*-test indicates a highly significant difference between DPPHb and FRAPb values (*t* = −8.67, *p* < 0.0001). FRAPb values are systematically higher, showing that the FRAP assay measured stronger antioxidant capacity in tomato extracts. The Pearson correlation between DPPHb and FRAPb is very strong (*r* = 0.96, *p* < 0.0001). Both assays are highly consistent and rank tomato cultivars in nearly the same order of antioxidant strength.

We assessed antioxidant activity in nine samples from eight tomato cultivars grown in the Czech Republic and six imported tomato samples available on the domestic market (Table S5). The antioxidant activity was determined by the DPPH and FRAP methods in plant material samples. The reported values represent the mean of three parallel measurements. For both methods, the results are expressed as milligrams of Trolox equivalents (TE) per gram of fresh weight, or per gram of dry matter for samples (Figure 6).

The antioxidant activity values of methanolic extracts from tomato cultivars grown in the Czech Republic or available on the Czech market (Table S5), determined by the DPPH and FRAP methods, ranged from 0.35 to 0.59 mg TE/g and from 0.50 to 1.12 mg TE/g, respectively (Figure 6A). The antioxidant activity measured by the DPPH method increased in the following order: Strabena, Karkulka < Brioso < Nelinka, Rubín < Bamano < Sweetelle 1, Sweetelle 2. The antioxidant activity determined by the FRAP method increased in the following order: Brioso, Karkulka < Bamano, Juanita, Nelinka, Rubín < Sweetelle 1 < Sweetelle 2. For the set of tomatoes from foreign production, the values of the antioxidant activity (VAA) obtained by the DPPH and FRAP methods ranged from 0.420 to 0.625 mg TE/g and from 0.677 to 1.175 mg TE/g, respectively. The results of antioxidant activity determined by the DPPH method were higher for the samples “tomatoes NL,” “Mini Roma,” and “Chockmato” than those for tomatoes grown in the Czech Republic. For VAA results measured by the FRAP method, no statistically significant difference was found between the cultivars richest in antioxidants grown domestically or abroad. The statistically significant lowest VAA values determined by the DPPH method were found for the cultivars Strabena and Karkulka, while for the FRAP method, the lowest values were recorded for Brioso and Karkulka (Figure 6B). It is evident that the VAA values determined by the DPPH method were statistically significantly lower for all tomato cultivars compared to the VAA values obtained by the FRAP method. Regression analysis revealed a strong linear correlation between the antioxidant activity results obtained by the DPPH and FRAP methods for tomato samples grown in the Czech Republic (correlation coefficient *r* = 0.9795; n = 9).

#### 3.6.2. Quercetin from Garlic and Other Natural Sources

In addition to conventional plant sources, we also explored the potential of non-traditional food materials, particularly vegetable waste, as alternative reservoirs of quercetin and other antioxidants. Within a circular-economy framework—aiming to maximize the value of raw materials and minimize waste—vegetable by-products rich in phenolic compounds can be transformed into high-value functional ingredients. To this end, ultrasound-assisted aqueous extraction (20 kHz) was applied to vegetable waste, yielding significantly higher extraction efficiency, shorter processing times, reduced energy consumption, and milder conditions compared with conventional boiling. The resulting extract exhibited antioxidant activities of 75.57 mg TE/g (DPPH) and 78.93 mg TE/g (FRAP), with a quercetin content of 4.3 mg/100 mL. The extract was subsequently fermented by a consortium of yeasts and bacteria at 23 °C for six days, producing a stable, non-alcoholic beverage with enhanced antioxidant capacity.

**Figure 7:**
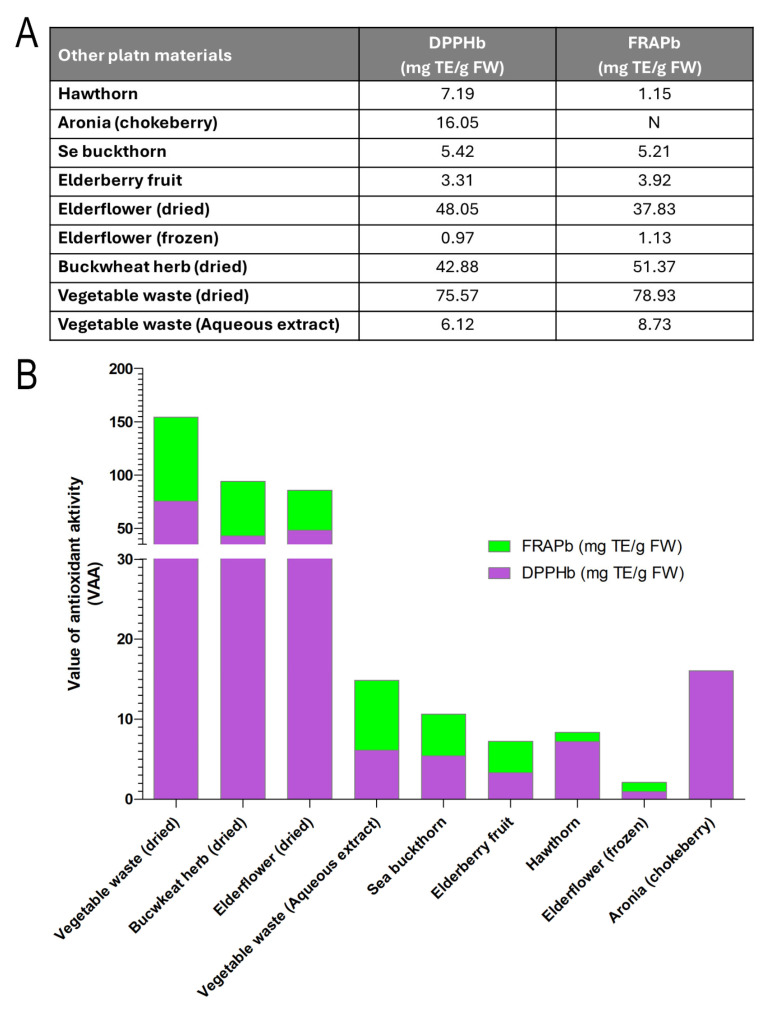
Antioxidant activity of selected plant materials determined by DPPH and FRAP assays. (**A**) Table showing values of antioxidant activity expressed as mg Trolox equivalents per gram of fresh weight (mg TE/g FW) for hawthorn, chokeberry, sea buckthorn, elderberry (fruit and flower), buckwheat herb, and vegetable waste (dried and aqueous extract). Unit is indicated as (mg TE/g FW), where c states for normalized concentration. (**B**) Bar chart comparing the results of the DPPHb (purple) and FRAPb (green) assays. Dried vegetable waste, buckwheat herb, and dried elderflower exhibited the highest antioxidant activities, while frozen elderflower and elderberry fruit showed the lowest. Statistical analysis of the results shows a very strong positive correlation between both methods (Pearson’s *r* = 0.981, *p* = 1.75 × 10^−5^), indicating high consistency in measured antioxidant potential. A paired *t*-test revealed no statistically significant difference between DPPH and FRAP values (*t* = 0.076, *p* = 0.941), confirming comparable outcomes of both analytical approaches.

The results revealed distinct differences between sources: dried vegetable waste, dried buckwheat herb, and dried elderflower displayed the highest antioxidant activities, whereas frozen elderflower and elderberry fruit showed the lowest. These findings demonstrate that both traditional wild-gathered plants (e.g., elderflower, buckwheat) and food-industry by-products (e.g., vegetable waste) can serve as meaningful sources of quercetin and other antioxidants. This not only underscores the nutritional potential of conventional botanical materials but also highlights the valorization of food-processing residues as promising raw materials for novel functional food ingredients (Figure 7).

The traditional source of quercetin is garlic. Detailed results on garlic cultivars grown in the Czech Republic are provided in the Supplementary Information (Figure S4). There, various aging conditions (temperature, time, and humidity) were tested, and aged garlic consistently showed markedly higher antioxidant activity compared to fresh garlic. The ranking of cultivars by combined antioxidant activity (VAA) was as follows: Havran ˃ Lan ˃ Bjetin ˃ Vekan ˃ Rusák ˃ Ivan, Lukan. Interestingly, while aged Havran scored highest in terms of antioxidant activity, cultivars such as Rusák and Bjetin received the best sensory evaluation.

In conclusion, our study provides experimental evidence that selected dietary antioxidants—particularly quercetin, lycopene, curcumin, and resveratrol—can modulate cellular stress responses and contribute to radioprotection by influencing IER5 activation and DNA repair pathways. We further demonstrated that their bioactivity depends not only on individual compound properties but also on their specific combinations, with some mixtures showing synergistic while others antagonistic effects. In addition, analyses of commonly consumed foods on the Czech market revealed marked differences in antioxidant potential across cultivars, underscoring the importance of food source and preparation in achieving protective effects. Together, these findings highlight the translational potential of dietary antioxidants for supporting genomic stability and resilience to ionizing radiation, with broader implications for preventive strategies in healthy aging. Future work should extend these observations into in vivo models and evaluate practical dietary interventions that can bridge the gap between experimental efficacy and population-level nutrition.

## 4. Discussion

### 4.1. Potential of Combinatory Antioxidants for Healthy Aging

In the context of an aging Czech and European population, maintaining genomic stability through diet may represent one of the key strategies to support healthy aging and reduce the burden of age-related diseases. However, despite the availability of cultivars rich in bioactive compounds such as lycopene and quercetin, the Czech population shows one of the lowest average vegetable intakes within the European Union. This discrepancy underscores a critical gap between potential dietary benefits and real-world consumption patterns. Increasing the intake of antioxidant-rich vegetables could therefore represent an accessible and preventive approach to improve resilience against oxidative stress and radiation-induced DNA damage in the broader population.

Quercetin, lycopene, curcumin, and resveratrol represent a group of bioactive compounds with complementary antioxidant and DNA-protective properties. Each of them has shown the ability to reduce oxidative stress, modulate stress-response pathways, and promote DNA repair in human cell models. When considered together, their diverse mechanisms suggest potential for synergistic interactions. Such combinations could provide broader protection against radiation-induced cellular damage and help to preserve genomic stability over time. This multifaceted activity highlights their promise as combinatory antioxidants that may contribute to healthy aging when incorporated into dietary strategies or formulated mixtures.

#### 4.1.1. Quercetin

In human cell models, quercetin pretreatment can significantly reduce radiation-induced DNA damage. For example, lymphocytes exposed to 2 Gy γ-irradiation showed fewer DNA strand breaks (lower comet tail DNA and micronucleus formation) when treated with quercetin prior to irradiation [58]. In vivo, quercetin also mitigated radiation injury to intestinal epithelium: mice given quercetin before abdominal X-ray exposure had improved survival, less crypt cell apoptosis, and accelerated post-irradiation tissue regeneration [59]. There is also evidence that quercetin can modulate DNA damage response signaling; in radioresistant cancer cells, quercetin was shown to activate and promote p53 [60], although in normal tissues, its activation of p53 may contribute to cell-cycle arrest and DNA repair. Direct data on quercetin’s influence on IER5 and γH2AX expression activation are limited. However, due to our results, regular involvement of quercetin food sources in daily consumption may be beneficial for healthy aging, reducing DNA damage (especially in lymphocytes and epithelial cells) by scavenging reactive oxygen species and enhancing endogenous antioxidant systems [61,62]. Taken all together, quercetin helps to not only lower the burden of radiation-induced lesions but also supports genomic stability, mechanisms that may contribute to healthier aging.

#### 4.1.2. Lycopene

Lycopene has demonstrated notable radioprotection in normal cell systems. Human epithelial and hematopoietic cells pretreated with lycopene are more resistant to radiation-induced genotoxicity. Gajowik et al. showed that exposing human lymphocytes to lycopene (10–40 μM) before irradiation significantly decreased DNA damage, as measured by comet assay tail moment and DNA in tail [63]. The greatest protection was seen when lycopene was present during irradiation; conversely, adding lycopene after exposure did not enhance DNA repair, highlighting that its main mode of action is preventive (i.e., reducing initial damage) rather than curative [63]. Lycopene also helps maintain endogenous antioxidant enzyme levels under stress, thereby reinforcing the cell’s own defense (systemsciencedirect.comsciencedirect.com). In summary, lycopene acts as a radioprotective agent chiefly through antioxidant mechanisms—minimizing initial oxidative DNA damage and attenuating the downstream DNA damage response. While specific effects of lycopene on early stress genes such as IER5 have not been reported, its capacity to reduce ATM/ATR activation suggests it may indirectly modulate early response gene expression by limiting the DNA damage signals that drive those genes. Biological availability of micronized lycopene gives opportunity to increase intake of this beneficial molecule even without need of frequent consumption of tomatoes in fresh or dried states, avoiding degradation of the molecule by heat preparation, such as cooking. Similar to quercetin, it may under specific conditions affect aging and help to prevent DNA damage. Indeed, several studies have shown that lycopene supplementation protects human lymphocytes from ionizing radiation by reducing DNA strand breaks and oxidative lesions [64]. By reducing the initiation of oxidative DNA damage and stabilizing endogenous antioxidant defenses, lycopene contributes to maintaining genomic integrity, which is an essential factor in healthy aging.

#### 4.1.3. Curcumin and Resveratrol

These two molecules were recognized as potential molecules which protect cells from irradiation and have a significant effect on the IER5- and γH2AX-activation pathways. Curcumin, the polyphenolic compound from turmeric, has been widely studied as both a radiosensitizer in tumors and a radioprotector in normal cell [37]. In human epithelial models and blood cells, curcumin protects against ionizing radiation by reducing oxidative DNA damage and enhancing repair. Curcumin not only directly neutralizes free radicals but also induces endogenous antioxidant defenses; it has been shown to elevate enzymatic antioxidants and glutathione, thereby mitigating radiation-induced oxidative stress [65]. Such effects translate into lower DNA damage (fewer micronuclei and chromosomal aberrations) and improved tissue integrity after radiation [66,67]. Overall, curcumin’s radioprotective profile is largely due to its antioxidative and anti-inflammatory properties, which reduce initial DNA damage and create a cellular environment conducive to effective DNA repair. These properties make curcumin a promising agent to protect human epithelial tissues from radiation toxicity.

In the case of resveratrol, it appears as both a radiosensitizer in cancer cells and a radioprotector in normal cells. Focusing on normal (non-malignant) epithelial models, resveratrol shows the ability to enhance DNA repair and antioxidant responses after radiation exposure. Resveratrol-treated cells formed more colonies after radiation and displayed fewer γ-H2AX foci and shorter comet assay tail moments, indicating reduced double-strand-break levels [68,69]. This suggests that resveratrol can both protect DNA from initial damage and/or facilitate more efficient repair. It also influences key damage-response proteins; for example, resveratrol has been shown to stabilize p53 in irradiated cells and to modulate cell-cycle checkpoints, allowing normal cells to recover faster from radiation-induced arrest [70]. Notably, mouse studies demonstrated that resveratrol given before and after radiation exposure reduced chromosomal aberration frequencies in bone marrow to near-background levels without increasing mutation rates [70]. This finding is in contrast to our observation; it implies that resveratrol accelerates accurate DNA repair, while our observation shows decreased activation of IER5 expression which points mostly to the protective effect of resveratrol in correlation with slightly increased γH2AX expression. This was relevant for the lower concentration of the resveratrol added to the cells prior to irradiation.

### 4.2. Availability of Consumed Foods on the European Market

From a broader perspective, the potential contribution of quercetin, lycopene, and potentially curcumin and resveratrol to healthy aging is especially relevant in the context of the Czech and wider European population, where demographic trends point to steadily increasing life expectancy but also a rising burden of age-related chronic diseases. Dietary patterns in Central Europe are often characterized by a limited intake of fresh fruits, vegetables, and spices rich in these bioactive compounds, while traditional preparation methods such as cooking or frying may further reduce their bioavailability. The supplementation or deliberate inclusion of foods naturally rich in these antioxidants could therefore help to mitigate cumulative oxidative damage and support the maintenance of genomic stability across lifespans. Although these molecules alone cannot counteract all determinants of aging, their combined incorporation into dietary strategies may contribute to delaying the onset of degenerative conditions, preserving functional capacity in older adults, and ultimately improving quality of life in aging societies.

Taken together, our results demonstrate that selected dietary antioxidants, when applied as single compounds or in carefully designed mixtures, can significantly influence IER5 expression and thereby modulate DNA repair responses in human epithelial cells. This mechanistic insight provides a biological basis for the observed health benefits associated with quercetin, curcumin, lycopene, and resveratrol, and supports their consideration not only as individual bioactive molecules but also as combinatory agents capable of synergistic activity. In the context of an aging Czech and European population, where maintaining genomic stability and resilience to environmental stressors is critical for healthy aging, such findings underscore the value of integrating these compounds into dietary strategies or functional formulations. By linking experimental evidence with societal needs, our study highlights the translational potential of radioprotective antioxidants in supporting longevity and quality of life.

## 5. Conclusions

This study demonstrates that selected dietary antioxidants, including quercetin, curcumin, lycopene, and resveratrol, exert measurable radioprotective effects in human epithelial cells. Their activity was evident not only as single compounds but also within defined mixtures, where synergistic or antagonistic interactions determined the overall impact on IER5 expression and DNA repair responses. The observed variability between individual foods and cultivars available on the Czech market further highlights the importance of source selection when considering natural carriers of bioactive compounds. Taken together, our findings provide mechanistic support for the role of these phytochemicals as modulators of radiation response and suggest their potential contribution to strategies promoting genomic stability and healthy aging. Future research should aim to validate these effects in vivo and to explore practical approaches for their incorporation into dietary patterns or functional formulations relevant for aging populations.

## Data Availability

The original contributions presented in this study are included in the article/Supplementary Materials. Further inquiries can be directed to the corresponding author.

## Supplementary Materials

The following supporting information can be downloaded at: https://www.mdpi.com/article/10.3390/antiox14111357/s1, Table S1: List of the components commercially available as food supplements; Table S2: Specification of the concentrations of supplements added to the cultivated cells; Figure S1: Graph of the measurement of the micronizes particles of each compound; Table S3: Statistical analysis of qPCR data showing differences in mRNA expression levels in response to varying concentrations of the tested compounds added to the cells; Figure S2: Correlation of the relative quantification of IER5 and γH2AX in supplemented cells after irradiation. Quercetin and lycopene effectively suppressed γH2AX with low abundance for activation of IER5, showing their potent radioprotective capacity; Table S4: Relationship between IER5 and γH2AX expression; Figure S3: Relationship between IER5 and γH2AX expression after irradiation; Table S5: Specifications of analyzed tomato cultivars; Figure S4: Antioxidant capacity of garlic cultivars grown in the Czech Republic. References [71,72,73,74,75] are cited in the Supplementary Materials.

## Abbreviations

The following abbreviations are used in this manuscript:

IER5	Immediate early response 5
ROS	Reactive oxygen species
DSBs	DNA double-strand breaks
hTERT RPE-1	Human retinal pigment epithelial cell line
DMEM	Dulbecco’s modified eagle medium
FBS	Fetal bovine serum
DPPH	2,2-diphenyl-1-picrylhydrazyl
FRAP	Ferric reducing antioxidant power
Fe^3+^-TPTZ	Ferric-tripyridyl triazine (Fe^3+^-TPTZ)
NF-κB	Nuclear factor-kappa B
NRF2	Nuclear factor erythroid 2-related factor 2
Gy	Gray
γH2AX	Phosphorylated form of histone variant H2AX
SOD	Superoxide Dismutase
p53	Tumor protein p53
UV	Ultraviolet radiation
UVB	Ultraviolet B radiation
VAA	Combined antioxidant activity
ATM	Ataxia-telangiectasia mutated
ATR	Ataxia-telangiectasia and Rad3-related protein
53BP1	Tumor suppressor p53-binding protein 1
GSH	γ-l-glutamyl-l-cysteinyl-glycine
ARE	Antioxidant response element
AMPK	Adenosine monophosphate-activated protein kinase
SIRT7	Sirtuin 7
HMGB1	High-mobility group box 1
