# Supplementary material for: Dietary Antioxidants Influence IER5 Activation and DNA Repair: Implications for Radioprotection and Healthy Aging
Source: Antioxidants (Basel). 2025 Nov 13;14(11):1357. doi: 10.3390/antiox14111357 (PMC12649379; doi:10.3390/antiox14111357)
Supplement: Supplementary file 1 [file antioxidants-14-01357-s001.zip › antioxidants-3892924-supplementary.pdf]

Table S1. List of the components commercially available as food supplements.

Name	Producer	Containt of the backage
Quercetin	GreenFood Nutrition	90 capsules
Betacarotene 10 000 IU	Jamieson	90 capsules
Curcumin 550 mg	Jamieson	30 capsules
Lycopene	Jamieson	60 tablets
Resveratrol 50 mg<br>*(Red vine extract)*	Jamieson	30 capsules
Lutein-Z	Jamieson	30 capsules
Kelp	Jamieson	90 tablets

Table S2. Specification of the concnetrations of suplements added to the cultivated cells.

Compound	Stock solution	Recomended concentrations in cultivation medium	*Reference*
**Quercetin**	50 mM (15 mg/ml)	50 µM	[1]
**Betacarotene**	10.000 IU v 500 µl + 0.1% Tween	1000 IU	[2]
**Curcumin**	100 mg/ml	100 µg/ml	[3]
**Resveratrol**	10 mM	10 µM	[4]
**Lycopene**	10 mg/ml + 0.1% Tween	5 µg/ml	[5]
**Lutein-Z**	10 mg/ml	10 µg/ml	[6]
**Kelp**	10 mM	10 µM	[7]

Figure S1

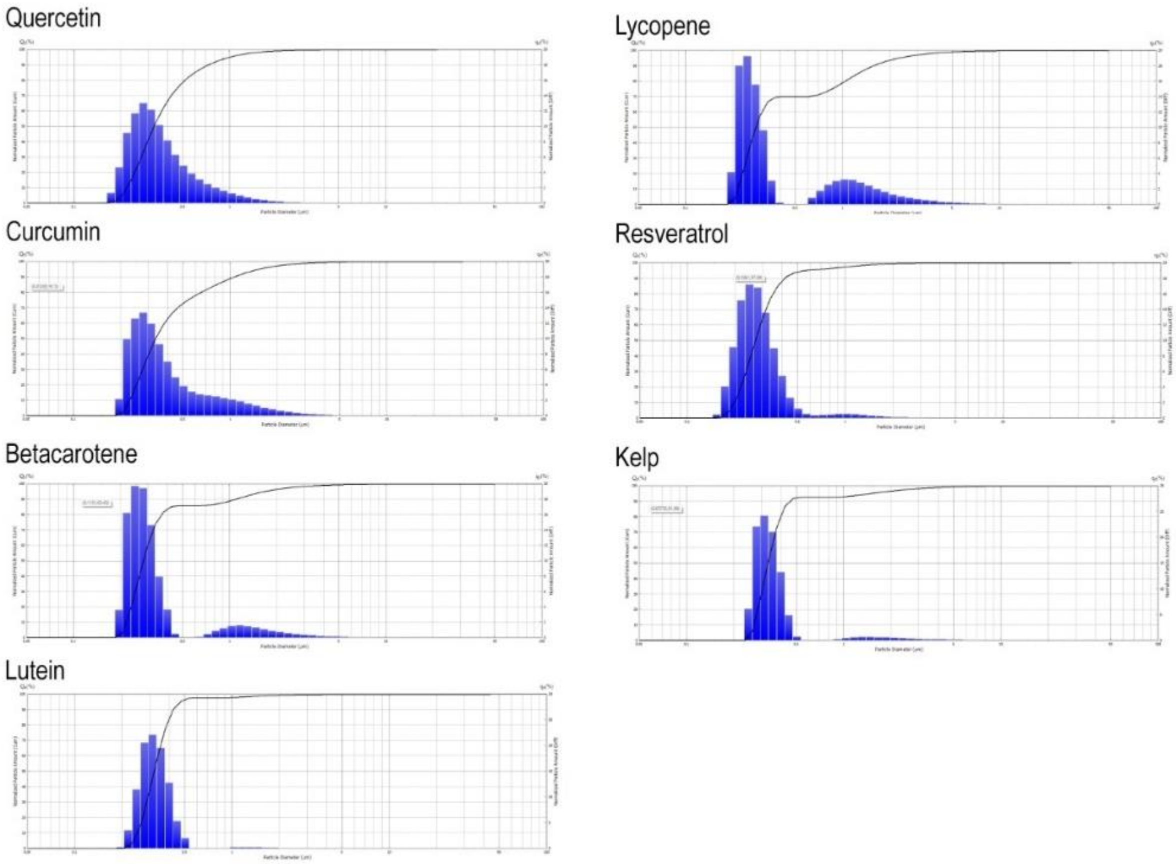

Figure S1. Graph of the measurement of the micronizes particles of each compound.

Table S3. Statistical analysis of qPCR data showing differences in mRNA expression levels in response to varying concentrations of the tested compounds added to the cells.

Compound	Comparison	Mean	Mean (diff)	p-value (t-test)	Significance
**Quercetin**	5 vs 50	1.02E-05	4.75E-08	0.837013972	ns
**Quercetin**	50 vs 100	1.01E-05	-0.044287658	8.38E-05	***
**Quercetin**	5 vs 100	1.02E-05	-0.044287611	8.38E-05	***
**Kurkumin**	5 vs 50	0.023758074	0.020054335	0.000401112	***
**Kurkumin**	50 vs 100	0.003703739	0.003693757	0.005085776	**
**Kurkumin**	5 vs 100	0.023758074	0.023748092	0.00029595	***
**B-karoten**	5 vs 50	0.12770163	0.106812689	6.46E-10	***
**B-karoten**	50 vs 100	0.020888942	-0.024431213	9.01E-07	***
**B-karoten**	5 vs 100	0.12770163	0.082381476	1.20E-07	***
**Lutein**	5 vs 50	0.052488799	-0.02211436	0.426544761	ns
**Lutein**	50 vs 100	0.074603159	0.012214757	0.702798762	ns
**Lutein**	5 vs 100	0.052488799	-0.009899603	0.671595603	ns
**Lykopen**	5 vs 50	0.028419497	0.028409564	0.030750745	*
**Lykopen**	50 vs 100	9.93E-06	-0.061134156	0.072566057	ns
**Lykopen**	5 vs 100	0.028419497	-0.032724592	0.29431754	ns
**Resvratrol**	5 vs 50	9.85E-06	-0.021233497	0.000170765	***
**Resvratrol**	50 vs 100	0.021243346	0.013558127	0.007874784	**
**Resvratrol**	5 vs 100	9.85E-06	-0.00767537	0.067866195	ns
**Kelp**	5 vs 50	0.029490405	0.001620956	0.77926781	ns
**Kelp**	50 vs 100	0.02786945	0.024066551	2.53E-11	***
**Kelp**	5 vs 100	0.029490405	0.025687507	0.005161019	**

Figure S2

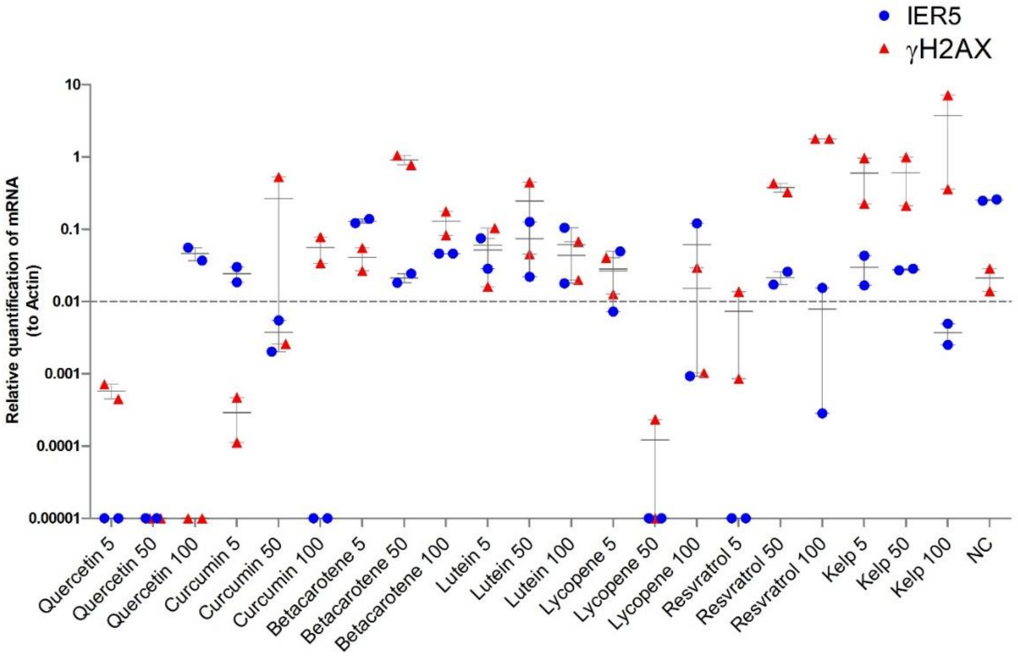

Figure S2. Correlation of the relative quantification of IER5 and γH2AX in supplemented cells after irradiation. Quercetin and lycopene effectively suppressed γH2AX with low abundance for acitvation of IER5, showing their potent radioprotective capacity.

Table S4. Relationship between IER5 and γH2AX expression. Regression analysis showing the correlation between *IER5* and γH2AX transcript levels ($\log_{10}$-transformed relative expression), with values from the non-treated control excluded.

Compound	Dosage	$\log_{10}$(IER5)	$\log_{10}$(γH2AX)
**Quercetin**	5	−5.00	−3.23
	50	−5.00	−5.00
	100	−1.33	−5.00
**Curcumin**	5	−1.62	−3.72
	50	−2.43	−0.58
	100	−5.00	−1.25
**Betacarotene**	5	−0.89	−1.39
	50	−1.67	−0.04
	100	−1.34	−0.99
**Lutein**	5	−1.29	−1.28
	50	−1.13	−0.61
	100	−1.21	−1.36
**Lycopene**	5	−1.55	−1.69
	50	−5.00	−3.74
	100	−1.22	−1.82
**Resveratrol**	5	−5.00	−2.23
	50	−1.67	−0.46
	100	−2.10	0.25
**Kelp**	5	−1.53	−0.30
	50	−1.56	−0.30
	100	−2.43	0.58

Pearson correlation (r): 0.42

p-value (two-tailed): 0.048

Spearman correlation (ρ): 0.46

p-value: 0.032

Regression line: $y = 0.46\,x - 0.50$

R^2: 0.19

Figure S3

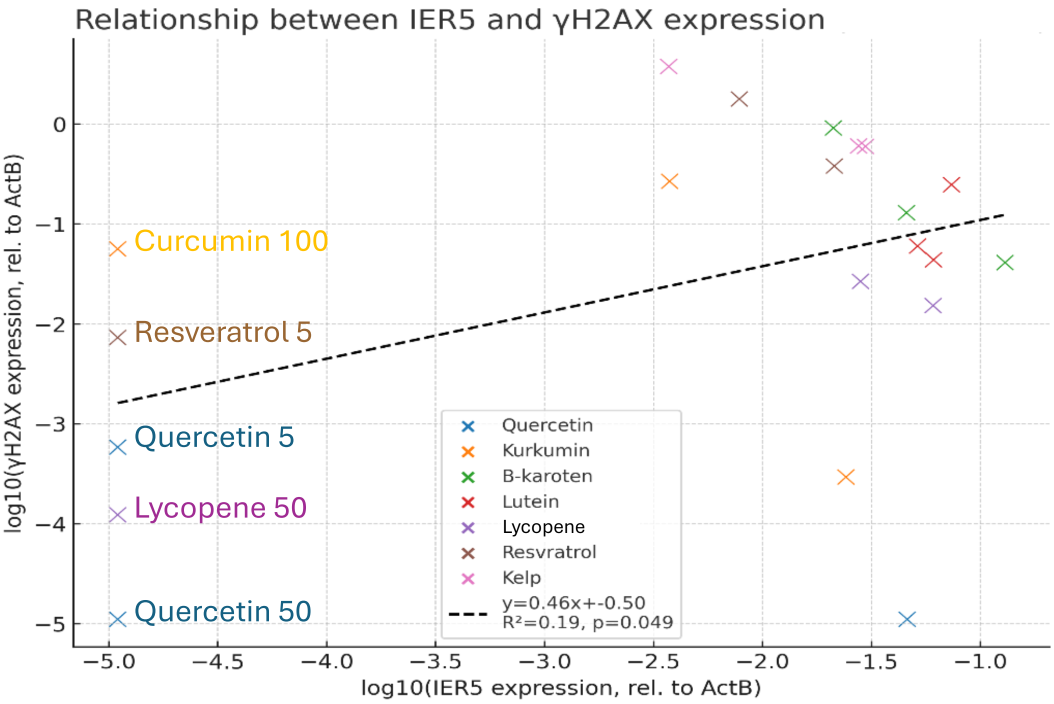

Figure S3. Relationship between *IER5* and γH2AX expression after irradiation. The scatter plot illustrates the relationship between *IER5* (x-axis) and γH2AX expression (y-axis) in cells exposed to γ-radiation and co-treated with individual radioprotective compounds. Each point represents the mean expression value (log_{10}-transformed relative to *ActB*), color-coded by compound as indicated in the legend. A moderate positive correlation was observed between *IER5* and γH2AX (Pearson p = 0.049; Spearman ρ = 0.46), with the regression line (black dashed) indicating that higher *IER5* transcription tends to accompany increased γH2AX signal intensity. The model explained approximately 19 % of the total variance (R^2 = 0.19), suggesting that while both markers are functionally linked, their responses to radiation and antioxidant treatments are only partially coupled.

Table S5. Specifications of analyzed tomato cultivars, showing name, size of the fruits, its colors, company which supply the cultivar on the market, and batch code of the tested samples.

Cultivar name	*Size grade*	*Color*	*Country of origin*	*Marketing cooperative brand*	*Batch code*
Bamano	20-30 mm	Orange	CZ	Čerozfrucht, s.r.o.	5714206
Brioso	35-45 mm	Red	CZ	Čerozfrucht, s.r.o.	L:2201
Juanita	25-30 mm	Red	CZ	Čerozfrucht, s.r.o.	L:2107
Karkulka	47-67 mm	Red	CZ	Čerstvě utrženo	N
Nelinka	21-35 mm	Red	CZ	Čerstvě utrženo	N
Rubín	25-35 mm	Red	CZ	Česká farma	5714226
Sweetelle 1	20-25 mm	Red	CZ	Čerozfrucht, s.r.o.	5715067
Sweetelle 2	23-35 mm	Red	CZ	Albertova tržnice	5713181
Strabena	23-35 mm	Red	CZ	Čerstvě utrženo	N
Chica	N	Red	ES	N	N
Chockmande	N	Brown	ES	N	N
Chockmato	N	Brown	ES	N	N
Mini Roma	N	Red	MA	N	1023097204534S0 B

Figure S4

A

Cultivar	Country of origin [a]	DPPHb (mg TE/g FW) [c]	FRAPb (mg TE/g FW) [c]
**Havran**	CR	8.19	8.25
**Lan**	CR	9.03	8.73
**Lukan**	CR	6.21	6.62
**Ivan**	CR	7.42	7.35
**Vekan**	CR	8.42	8.22
**Rusák**	CR	8.42	8.29
**Bjetin**	CR	8.27	8.31

B

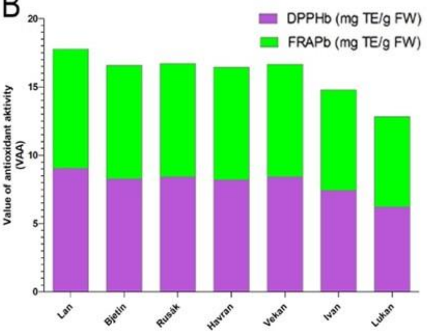

Figure S4. Antioxidant capacity of garlic cultivars grown in the Czech Republic. (A) Table showing values of antioxidant activity determined by the DPPH and FRAP assays for seven Czech cultivars. Data are expressed as mg Trolox equivalents per gram of fresh weight (mg TE·g^{-1} FW). **(B)** Bar graph comparing the values of antioxidant activity obtained by both methods for individual cultivars, ordered from the most effective to the less effective. The paired *t*-test comparing DPPH and FRAP assay results ($t = 0.31$, $p = 0.77$) showed no significant difference between the two analytical methods, indicating that both assays yielded statistically comparable estimates of antioxidant activity for garlic samples. In contrast, the Pearson correlation coefficient was very high ($r = 0.99$, $p < 0.0001$), demonstrating an extremely strong positive linear relationship between the DPPH and FRAP measurements.

References

1. Danihelová, M.; Veverka, M.; Šturdík, E.; Jantová, S. Antioxidant Action and Cytotoxicity on HeLa and NIH-3T3 Cells of New Quercetin Derivatives. *Interdiscip. Toxicol.* **2013**, *6*, 209–216, doi:10.2478/INTOX-2013-0031.
2. Konopacka, M.; Widel, M.; Rzeszowska-Wolny, J. Modifying Effect of Vitamins C, E and Beta-Carotene against Gamma-Ray-Induced DNA Damage in Mouse Cells. *Mutat. Res. - Genet. Toxicol. Environ. Mutagen.* **1998**, *417*, 85–94, doi:10.1016/S1383-5718(98)00095-3.
3. Zoi, V.; Galani, V.; Tsekeris, P.; Kyritsis, A.P.; Alexiou, G.A. Radiosensitization and Radioprotection by Curcumin in Glioblastoma and Other Cancers. *Biomedicines* **2022**, *10*, doi:10.3390/BIOMEDICINES10020312,.
4. Fukui, M.; Choi, H.J.; Zhu Bao Ting, B.T. Mechanism for the Protective Effect of Resveratrol against Oxidative Stress-Induced Neuronal Death. *Free Radic. Biol. Med.* **2010**, *49*, 800–813, doi:10.1016/j.freeradbiomed.2010.06.002.
5. Wahnou, H.; El Kebbaj, R.; Liagre, B.; Sol, V.; Limami, Y.; Duval, R.E. Curcumin-Based Nanoparticles: Advancements and Challenges in Tumor Therapy. *Pharm. 2025 Vol 17 Page 114* **2025**, *17*, 114, doi:10.3390/PHARMACEUTICS17010114.
6. Frede, K.; Ebert, F.; Kipp, A.P.; Schwerdtle, T.; Baldermann, S. Lutein Activates the Transcription Factor Nrf2 in Human Retinal Pigment Epithelial Cells. *J. Agric. Food Chem.* **2017**, *65*, 5944–5952, doi:10.1021/ACS.JAFC.7B01929,.
7. Stefaniak–Vidarsson, M.M.; Gudjónsdóttir, M.; Marteinsdottir, G.; Omarsdottir, S.; Bravo, E.; Sigurjonsson, O.E.; Kristbergsson, K. Determination of Bioactive Properties of Food Grade Extracts from Icelandic Edible Brown Seaweed Sugar Kelp (Saccharina Latissima) with in Vitro Human Cell Cultures (THP-1). *Funct. Foods Health Dis.* **2019**, *9*, 1–15, doi:10.31989/FFHD.V9I1.546.
